# Biomass composite with exogenous organic acid addition supports the growth of sweet sorghum (*Sorghum bicolor* ‘*Dochna*’) by reducing salinity and increasing nutrient levels in coastal saline–alkaline soil

**DOI:** 10.3389/fpls.2023.1163195

**Published:** 2023-03-28

**Authors:** Ruixue Yang, Zhengguo Sun, Xinbao Liu, Xiaohua Long, Limin Gao, Yixin Shen

**Affiliations:** ^1^ College of Agro-grassland Science, Nanjing Agricultural University, Nanjing, China; ^2^ College of Resources and Environmental Sciences, Nanjing Agricultural University, Nanjing, China; ^3^ Ecological Research Center, Nanjing Institute of Agricultural Sciences in Jiangsu Hilly Area, Nanjing, China

**Keywords:** sweet sorghum, coastal saline-alkali soil, exogenous organic acid, biomass material, saline-alkali regulation, soil fertility improvement, resistant growth

## Abstract

**Introduction:**

In coastal saline lands, organic matter is scarce and saline stress is high. Exploring the promotion effect of intervention with organic acid from biological materials on soil improvement and thus forage output and determining the related mechanism are beneficial to the potential cultivation and resourceful, high-value utilization of coastal mudflats as back-up arable land.

**Method:**

Three exogenous organic acids [humic acid (H), fulvic acid (F), and citric acid (C)] were combined with four kinds of biomass materials [cottonseed hull (CH), cow manure (CM), grass charcoal (GC), and pine needle (PN)] and applied to about 0.3% of medium-salt mudflat soil. The salinity and nutrient dynamics of the soil and the growth and physiological differences of sweet sorghum at the seedling, elongation, and heading stages were observed under different treatments to screen for efficient combinations and analyze the intrinsic causes and influencing mechanisms.

**Results:**

The soil salinity, nutrient dynamics, and forage grass biological yield during sweet sorghum cultivation in saline soils differed significantly (*p* < 0.05) depending on the type of organic acid–biomass composite applied. Citric acid–pine needle composite substantially reduced the soil salinity and increased the soil nutrient content at the seedling stage and improved the root vigor and photosynthesis of sweet sorghum by increasing its stress tolerance, allowing plant morphological restructuring for a high biological yield. The improvement effect of fulvic acid–pine needle or fulvic acid–cow manure composite was manifested at the elongation and heading stages.

**Discussion:**

Citric acid–pine needle composite promoted the growth of saline sweet sorghum seedlings, and the effect of fulvic acid–pine needle composite lasted until the middle and late stages.

## Introduction

Saline–alkaline soil has many limiting factors in agricultural production due to excessive salt enrichment and high levels of exchangeable sodium (Uri, 2018) and can be classified as primary or secondary saline according to its genesis (Negacz et al., 2022). Coastal saline soils are one of the main types of primary saline soils and are formed by the siltation of incoming sediment and impregnation by highly mineralized seawater (Cui et al., 2021). These lands are extremely widely distributed in the coastal areas of China, which involve 11 provinces or municipalities with a total coastline length of 3.2 × 10^4^ km and a total mudflat area of 1.3 × 10^7^ ha. The total area of mudflats in Jiangsu Province alone accounts for 6.52 × 10^5^ ha, which is about 25% of the total area of mudflats in China (Shao et al., 2019; Yang et al., 2021a; Zhang et al., 2020b). However, seed germination and crop seedling formation, establishment, and growth are greatly impaired due to the high degree of salinization, nutrient deficiency, low nutrient utilization (Wang et al., 2020b), and poor soil texture and structural stability of mudflat soils, and the seasonal salt regression and frequent salt accumulation (Odeh and Onus, 2008; Dong et al., 2020). In some cases, serious osmotic stress and ion toxicity even occur (Sun et al., 2022). Therefore, saline–alkaline soil is difficult to use directly because of the restrictive factors in agricultural production (Paul and Lade, 2014; Ranasinghe and Piyadasa, 2020). According to the current national marine economic development planning outline and other relevant documents, adopting scientific and efficient methods to manage saline–alkaline land is of great significance to maintain the red line of cultivated land, stabilize the cultivated land fertility, ensure food security, and efficiently use plant–land resources in a coastal wetland ecosystem. In addition, these techniques can be used to fully exploit the potential and orderly develop saline–alkaline land as reserve farmland and explore land management and utilization patterns in line with economic and social development in coastal areas.

The use of biological methods to improve saline soils is beneficial in the development of the virtuous cycle of the plant–soil feedback system under saline conditions (Meki et al., 2022), gradually eliminating saline components from the soil and improving soil nutrients to fully utilize them. Sweet sorghum (*Sorghum bicolor* ‘*Dochna*’) is a saline–alkaline-tolerant C4 plant with a long history of cultivation in coastal areas of eastern China, making it more suitable than other forage grasses for bioremediation activities in mudflat soils that lack freshwater resources (Shi et al., 2022; Zhang et al., 2023). Although planting salt-tolerant forage grasses as a biological improvement measure for saline soils has received recognition and support (Wang et al., 2018), medium- to high-salinity soils still cause great stress on the seed germination and seedling establishment of salt-tolerant forage grass. For this reason, biomass materials are usually introduced to provide a favorable soil environment in the root zone for salt-tolerant forage grasses to avoid the influence of high salt; the signal information and related substances of reducing salt, regulating acidity, and increasing fertility are simultaneously sent to the soil (El Hasini et al., 2019). Biomass materials refer to environmentally friendly materials obtained by further fermentation or heat treatment using industrial by-products or waste from the production of agriculture, forestry, and livestock as raw materials (Kulikova et al., 2022). Common biomass materials include crop straw, wood chips, cottonseed hulls, cow manure, pine needles, mushroom residue, and grass charcoal. These biomass materials have a high potential for saline soil improvement due to their structural stability and adsorption properties (Wang et al., 2017). Planting salt-tolerant forage grasses grown in saline soils with low-to-medium salinity could improve the physical and hydraulic properties of saline soils through biomass material addition (Liang et al., 2021) and regulate their nutrient status and functional microbial activity (Li and Li, 2022; Yao et al., 2022a). Biomass has a unique microporous structure and some functional groups that can cut off the continuity of soil capillaries, inhibit the transport and accumulation of water and salts to the upper layers of the soil (Wu et al., 2021c), and improve soil porosity and structure by forming iron complexes to increase soil aggregates (Li et al., 2021; Chen et al., 2022). In addition, the exogenous application of biomass increases the content of energy substrates and bioactive substances in soil (Kaur et al., 2005; Wong et al., 2009), significantly increases soil microbial and enzyme activities, and regulates soil microbial community structure (Wu et al., 2021a), thus favorably affecting plant root and aboveground growth and soil ecology (Chen et al., 2021b; Li et al., 2022a). However, the differences in composition and internal mechanism among biomass materials lead to their varying effects on the growth of salt-tolerant forage grasses in saline–alkaline soil and the improvement of the soil itself (Amini et al., 2016).

**Figure 1:**
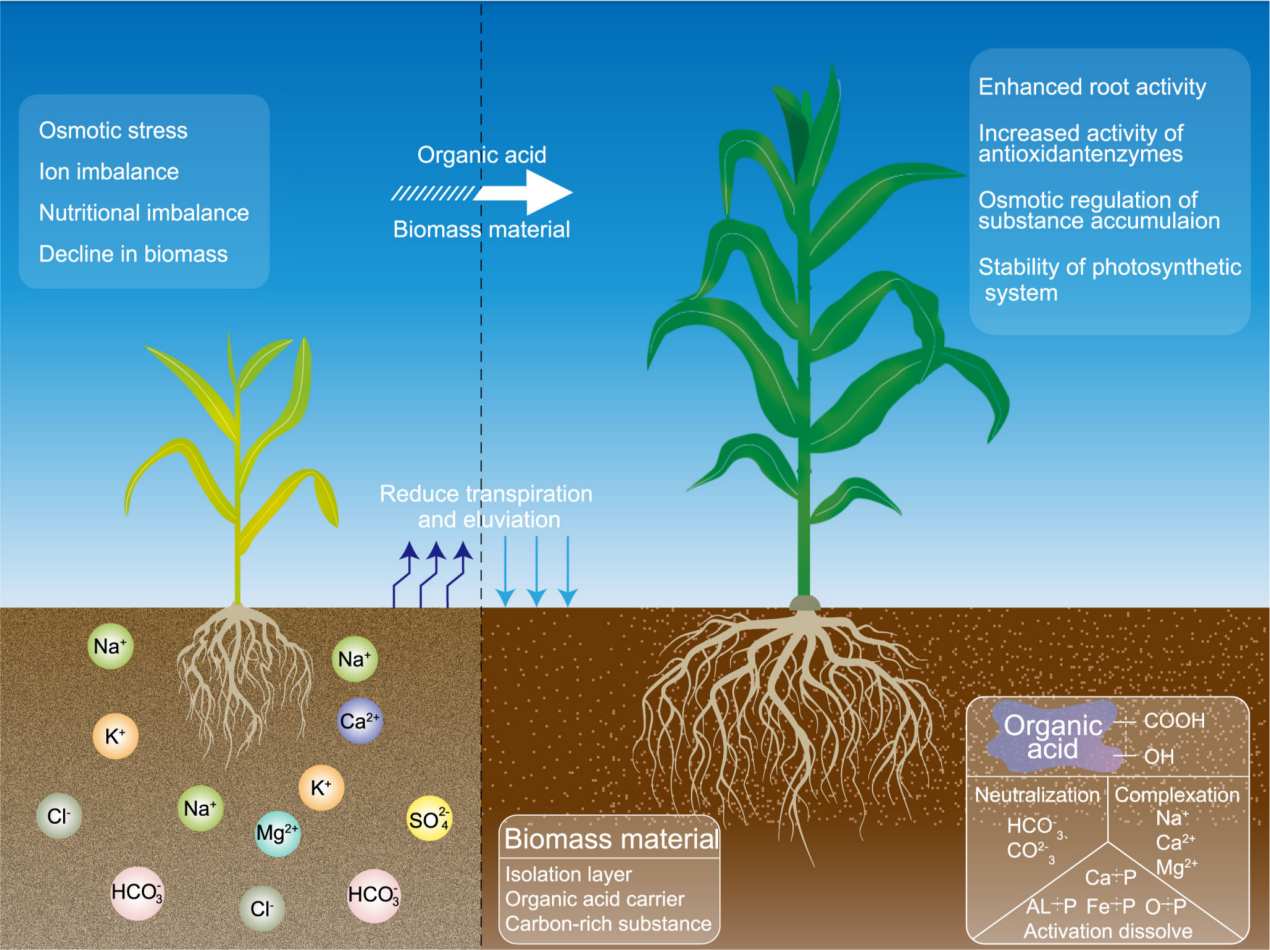
Model diagram of sweet sorghum growth improved by exogenous organic acid and biomass materials in coastal mudflat soil.

With the progressive understanding of the structure and function of organic acids, composites rich in citric acid, humic acid, fulvic acid, or other organic acid components have become new products in the fields of environmental remediation and crop growth regulation (Ma et al., 2020; Guo et al., 2022). These chemical conditioners, fertilizers, and other functional products whose main component is organic acid are referred to as organic acid products. The addition of organic acids and their products to saline soils can neutralize soil alkalinity through the acid hydrolysis of active functional groups, enhance the solubility and mobility of insoluble nutrients, and adsorb metal ions from saline–alkaline soil through complexation and chelation; as a result, the nutrient content and physical structure of the soil are further improved (Liu et al., 2020; Hu et al., 2021; Xiao et al., 2022); the rapid alkali reduction and desalting are achieved; the nutrient reservoir capacity of saline–alkaline land is increased; and the plant nutrient utilization is improved (Liu et al., 2019; Mosaad et al., 2022). It can also improve crop yield by changing the root architecture, increasing root vigor and growth, and intercepting additional nutrients from the soil by adjusting the density of primary and secondary roots and the number of branches (Liao et al., 2006; Aoki et al., 2012; Neumann et al., 2000; Zandonadi et al., 2007). It can help plants under saline stress to increase the ratio of K^+^/Na^+^ (Li et al., 2022b), enhance the accumulation of soluble sugar and soluble protein, increase the concentration of osmoregulatory substances and the function of the antioxidant system (Do Nascimento et al., 2006; Liu et al., 2015), alleviate lipid peroxidation, and improve the salt tolerance index of crops (Mallhi et al., 2019). Therefore, the above-mentioned biomass materials and organic acids or their products have stronger effects on salt-tolerant forage production and soil salinity reduction in saline soils. However, whether the combination of different types of organic acid with different types of biomass as a treatment for salt-tolerant forage grasses in saline soils is more effective than their individual effects at the seedling stage (when resistance is relatively weak), elongation stage (when growth is fast), and heading stage (when nutrients start to be transferred) is unknown. A comprehensive literature review revealed the lack of studies in this area. Therefore, this research proposed a technical model (**Figure 1**) for the exogenous application of organic acids combined with biomass for salt-tolerant forage in saline soils. The findings will broaden the perception of biomass methods for improving saline soils and serve as the basis for the application of organic acids or their products in saline soil agriculture.

For the saline–alkaline characteristics of mudflat soils and based on the physicochemical data of organic materials, three representative natural organic acids in soils, humic acid (H), fulvic acid (F), and citric acid (C), were selected with four acidic or neutral biomass materials with good structure, namely, cottonseed hull (CH), cow manure (CM), grass charcoal (GC), and pine needle (PN). Several exogenous organic acids were composited with different biomass materials and applied as a treatment for sweet sorghum growing in the coastal saline–alkaline land of eastern China to investigate their effects on plant production performance with respect to soil nutrients and salinity and to explore the mechanisms related to the interaction between different types of organic acids and biomass materials and its effect on soil improvement and alleviation of saline–alkaline stress in crops. The main objectives of this study were as follows: (i) to explore the mechanisms of the effects of different exogenous organic acid-biomass material composite on the salinity dynamics and nutrient accumulation in coastal mudflat soils; (ii) to determine the factor driving the relationship between different exogenous organic acid-biomass material composite with the growth, physiological changes, and accumulation of active compounds in sweet sorghum under saline stress; and (iii) to reveal the correlation between the physicochemical characteristics of mudflat soil and the growth performance of plants under treatments with different exogenous organic acid–biomass material composite. The results will help enhance the understanding of different types of exogenous organic acid–biomass composites for soil amendment or targeted improvement of plant stress tolerance and provide new ideas for organic acid utilization.

## Materials and methods

### Overview of the research area

The experiment was carried out in a coastal saline soil in the strip mud reclamation area of Jianggang Town, Dongtai City, Jiangsu Province, China (N 32° 83′, E 120° 96′). The soil type in this area is alluvial tidal saline soil, which belongs to silty loam and contains 20.5% sand, 8.0% clay, and 71.5% silt (0–20 cm). The specific physicochemical properties are shown in **Table 1**. The area has a subtropical monsoon maritime climate with an average annual temperature of 16.3°C and an average annual precipitation of 1,024 mm, which mainly occurs in summer.

**Table 1:** Basic physical and chemical properties of primitive soil.

Properties	Values	Properties	Values
Field water capacity (%)	23.05 ± 0.16	Organic matter content (g kg^−1^)	8.77 ± 0.02
Soil bulk density (g cm^−3^)	1.47 ± 0.04	Total nitrogen content (g kg^−1^)	0.45 ± 0.01
Soil pH	9.16 ± 0.01	Total phosphorus content (g kg^−1^)	0.52 ± 0.02
Soil electrical conductivity_1:5_ (μs cm^−1^)	492.0 ± 4.73	Alkali-hydrolyzed nitrogen content (mg kg^−1^)	22.18 ± 0.13
Soil soluble salt content (g kg^−1^)	3.22 ± 0.04	Available phosphorus content (mg kg^−1^)	14.18 ± 0.02

### Experimental design and field management

**Figure 2:**
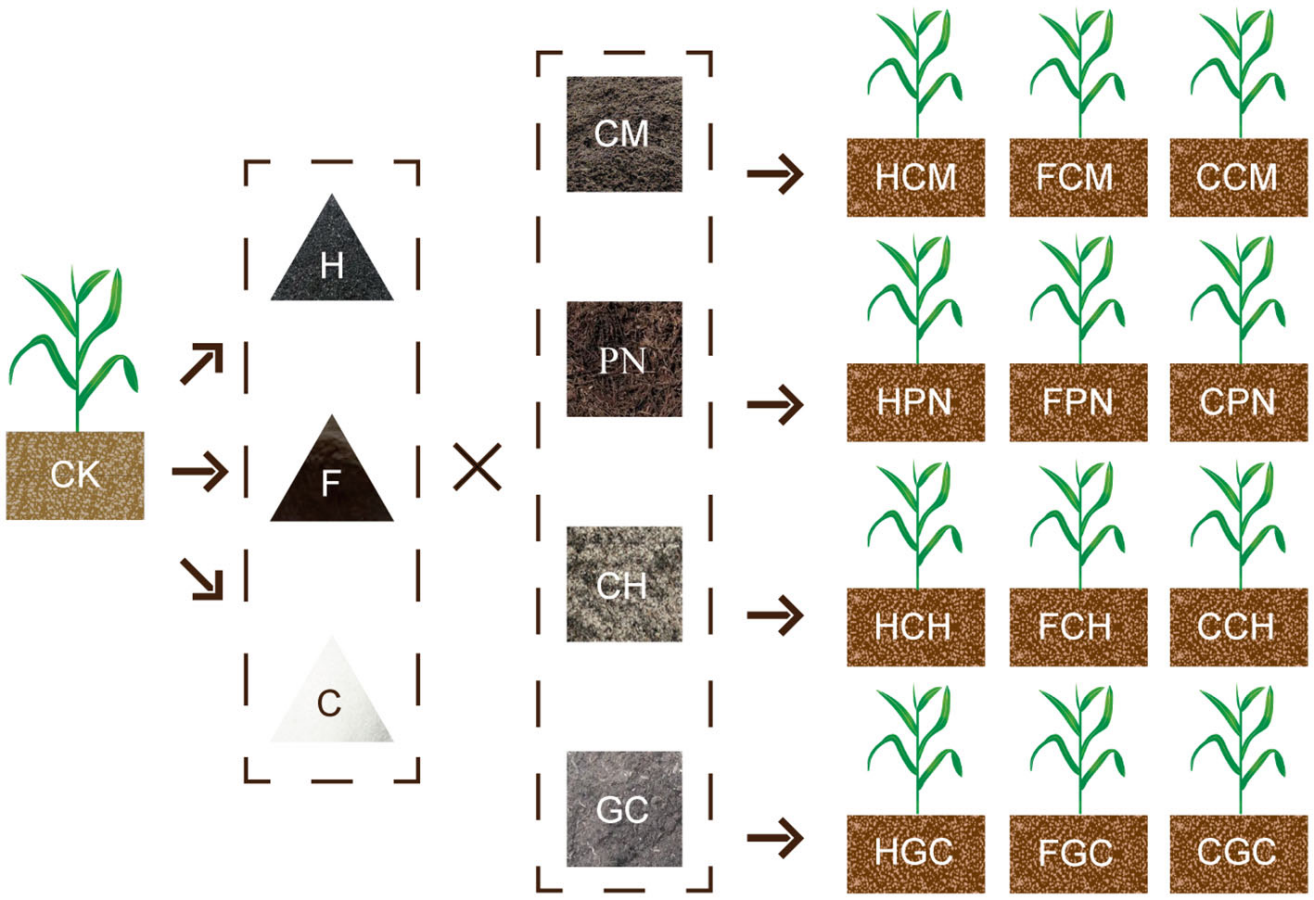
Schematic of the two-factor combination and treatment distribution. H, humic acid; F, fulvic acid; C, citric acid; CH, cottonseed hull; CM, cow manure; GC, grass charcoal; PN, pine needle; HCH, humic acid–cottonseed hull composite; HCM, humic acid–cow manure composite; HGC, humic acid–grass charcoal composite; HPN, humic acid–pine needle composite; FCH, fulvic acid–cottonseed hull composite; FCM, fulvic acid–cow manure composite; FGC, fulvic acid–grass charcoal composite; FPN, fulvic acid–pine needle composite; CCH, citric acid–cottonseed hull composite; CCM, citric acid–cow manure composite; CGC, citric acid–grass charcoal composite; CPN, citric acid–pine needle composite; CK, without the addition of organic acids and biomasses. The same as **Tables 2**, **3**, and **Figures 3** **–** **7**.

The field experiment was conducted on sweet sorghum samples under 13 treatments in June 2021 (**Figure 2**):(1) CK, (2) HCM, (3) HPN, (4) HCH, (5) HGC, (6) FCM, (7) FPN, (8) FCH, (9) FGC, (10) CCM, (11) CPN, (12) CCH, and (13) CGC. All treatments were replicated three times, and the application rates of organic acid and biomass were 120 and 5,000 kg ha^−1^, respectively, in all treatments. The cultivar of sweet sorghum used was “Big Kahuna,” a new forage sorghum variety that is drought and salinity tolerant and has photoperiod sensitivity and brown midrib characteristics, resulting in high biological yield and nutritional value. The sources and properties of organic acid materials are shown in **Table 2**. For the biomass materials, the CM was aerobically composted and fermented for about 40 days after wet and dry separation; the CH was made from the residual husk scraps of edible mushroom culture material; the GC was prepared from the accumulation of incompletely decomposed plant residues fermented in an overly wet and suspicious natural environment; and the PN were those that had accumulated on the soil surface of lacebark pine forests and collected after natural weathering and decomposition. The specific physical and chemical properties of these biomass materials are shown in **Table 3**. The study used a randomized block design with split plots, where organic acid was the main plot and biomass was the split plot, with a plot arrangement of 10 m^2^ (4 m long and 2.5 m wide). The soil tillage layer (0–20 cm) was rototilled before the start of the experiment. In addition to the land preparation, drainage ditches (30 cm deep and 40 cm wide) were opened between the plots so that these ditches could be connected to the drainage pipes at the edges of the fields to avoid seedling damage caused by waterlogging in the fields. A double layer of mulch was placed along a vertical depth of 0–30 cm close to the edge of each plot to stop the migration of water salts, nutrients, and other substances between the plots. The base fertilizer application was 300 kg ha^−1^ of compound fertilizer (N, 15%; P, 15%; and K, 15%), which was applied during land preparation. The follow-up fertilizer was applied twice at 30 and 60 days after seedling emergence with 75 kg ha^−1^ of urea (N, 46.4%). The organic acid pellets (or powder) were mixed evenly with the biomass material and then spread into the plots and pulled back and forth with a rake to mix evenly into the topsoil. The sweet sorghum variety was Big Kahuna, which was planted in June 2021 using the strip sowing method with a row spacing of 40 cm and a sowing depth of 2 cm. The seedlings were set to a specification standard of 25 cm apart during the three-leaf period. During the growing season, timely prevention and elimination were carried out in conjunction with the occurrence of diseases, insects, and weeds in the field. Owing to the abundant rainfall during the growing season, no irrigation was carried out for moisture management and the water in the field was removed in a timely manner.

**Table 2:** Sources and basic properties of organic acids.

Organic acid species	Solubility	Structure	Appearance	pH	C (%)	H (%)	O (%)	N (%)
H^a^	Slightly soluble in water	High molecular polymer	Black powder	6.82 ± 0.02	37.91 ± 0.24	3.46 ± 0.03	25.43 ± 0.24	0.61 ± 0.01
F^a^	Soluble in water, acid, alkali	High molecular polymer	Brown powder	5.26 ± 0.03	28.35 ± 0.18	4.23 ± 0.02	41.18 ± 0.17	3.24 ± 0.02
C^b^	Highly soluble in water	Low molecular weight	White granule	1.47 ± 0.01	36.72 ± 0.10	4.77 ± 0.03	55.30 ± 0.19	0.18 ± 0.01

a The H and F are provided by Xinjiang Shengda Yifang Biotechnology Co. Ltd.

b The C is purchased by Weifang Yingxuan Industrial Co. Ltd.

**Table 3:** Sources and basic properties of biomass materials.

Biologic material species	pH	Electrical conductivity_1:5_ (μs cm ^± 1^)	Organic matter content (g kg^−1^)	Total nitrogen content (g kg^−1^)	Total phosphorus content (g kg^−1^)	Alkali-hydrolyzed nitrogen content (mg kg^−1^)	Available phosphorus content (mg kg^−1^)
CH^a^	7.3 ± 0.03	1,682 ± 15.52	297.9 ± 3.58	11.85 ± 0.63	2.95 ± 0.01	728.9 ± 8.24	776.7 ± 8.98
PN^a^	5.38 ± 0.02	1,308 ± 6.66	407.9 ± 5.54	10.2 ± 0.09	0.66 ± 0.01	1,375 ± 24.71	79.73 ± 1.87
GC^a^	5.08 ± 0.03	841.0 ± 9.07	244.1 ± 3.22	8.46 ± 0.02	0.79 ± 0.01	693.2 ± 16.13	45.82 ± 1.75
CM^b^	7.70 ± 0.03	1,416 ± 8.14	345.8 ± 4.91	9.69 ± 0.04	2.36 ± 0.02	477.6 ± 6.69	519.8 ± 8.53

a The CH, PN, and GC are purchased by Shijiazhuang Nongyou Biotechnology Co. Ltd.

b The CM is provided by Jurong Lantian Bishui Biotechnology Co. Ltd.

### Plant and soil sampling

Samples were taken at 30 days (when the leaves reached five to seven and the plant was in the seedling stage of rapid seedling growth), 60 days (when the nodes can be touched at the base edge of the stem and the plant was in the elongation stage of rapid internode elongation), and 90 days (when the uppermost flag leaf of the plant was fully expanded and the plant was in the heading stage of nutrient transfer) after emergence. Dig a 30-cm-diameter and 40-cm-deep soil column centered on sweet sorghum and remove the soil attached to the root system to obtain a complete plant. Five representative plants from each plot were collected and placed in a low-temperature preservation box and brought back to the laboratory. Soil (0–20 cm) from each plot was sampled using the five-point method (“S” distribution) using a soil auger, placed in sterile, sealed bags, and brought back to the laboratory to air-dry for testing.

### Plant and soil analysis

The sweet sorghum was rinsed with water and then dried. The absolute length from the base of the plant stalk up to the absolute length of the highest point where the sword leaves were straightened was measured using a tape measure as plant height. The diameter at the middle of the first internode at the base of the plant stem was measured as stem diameter using vernier calipers. The absolute length of the plant from the base of the stem to the longest root position was measured as the root length, and the number of plant leaves was recorded. The above-ground part was then separated from the root system, heated in an oven at 105°C for 30 min, and then dried at 65°C to constant weight. The above-ground part and root system were weighed separately, and the whole plant’s dry weight was labeled as single plant biomass. Root activity was determined using the triphenyltetrazolium chloride method (Lassheikki et al., 1991). The procedure and method for the determination of chlorophyll content were referred to by Ghobadi et al. (2013). Malondialdehyde content was determined using the method of Wang and Jin (2005). The procedure and calculation of proline content were referred to by Bates et al. (1973). Soluble sugar content was determined by the modified method of Sun et al. (2015). Superoxide dismutase (SOD), peroxidase (POD), and catalase (CAT) activities were determined by the method of Raza et al. (2007). Soil organic matter content was determined by the wet digestion method using potassium dichromate reagent (Jones, 2017). Soil alkaline-hydrolyzed nitrogen content was analyzed by the diffusion absorption method, and soil available phosphorus content was determined by the colorimetric method referring to the study of Pansu and Gautheyrou (2006) for the specific analytical methods. Soil pH was determined using a pH meter (*Eutech* pH 700, USA) with a soil-to-water ratio of 1:5. Soil electrical conductivity was determined using a conductivity meter (DDS-307A, Shanghai, China) with a soil-to-water ratio of 1:5. The soil-soluble salt content was measured using the solid residue method of Makkaveev and Stunzhas (2017). After the relationship between soil soluble salt content and electrical conductivity was obtained, the soil soluble salt content of all samples was calculated according to the following formula:


SS=0.0068EC−0.1305,

Where SS means soil-soluble salt content (g·kg^−1^) and EC represents soil electrical conductivity (μs·cm^-1^)

### Statistical analysis

Microsoft Excel 2010 was used for preliminary statistics, and IBM SPSS Statistics 23.0 software was employed for one- and two-way ANOVA. Duncan’s test was applied for multiple comparisons of the data, and a two-way ANOVA was used to test two factors and their interactions. Origin 2022 was utilized to produce the graphs. Data were expressed as “mean ± standard error,” and significant differences were expressed using the alphabetical method (*p* < 0.05). Correlation plot software in Origin 2022 was used for correlation analysis.

## Results

### Effects of different combinations of exogenous organic acids and biomass materials on the saline–alkaline dynamics of mudflat soil

**Figure 3:**
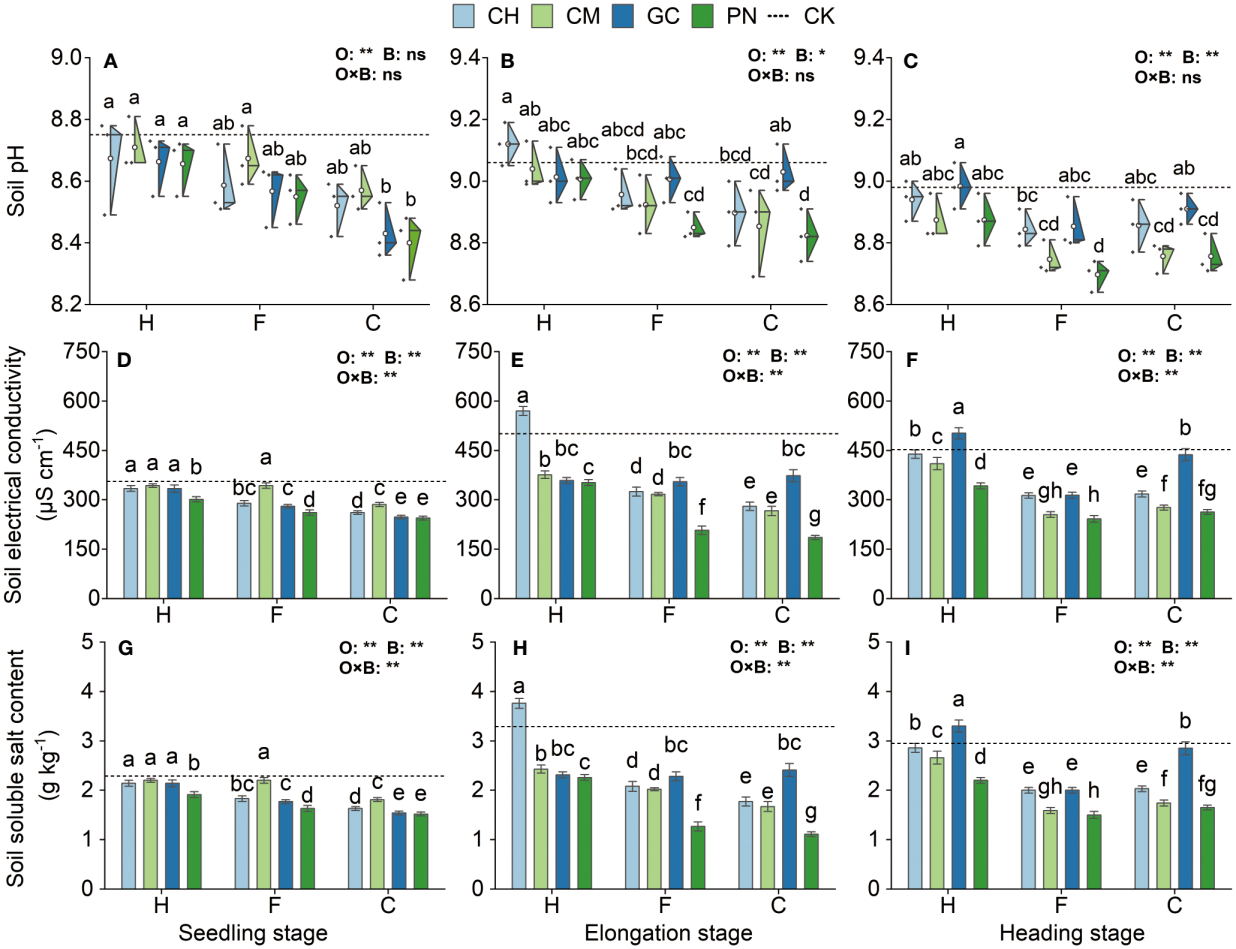
Effects of exogenous organic acids combined with biomass materials on the pH, electrical conductivity, and soluble salt content of mudflat soil. **(A, D, G)** Represent the seedling stage, **(B,E,H)** represent the elongation stage, and **(C, F, I)** indicate as the heading stage. O, organic acid treatment; B, biomass material treatment; O×B, combined treatment of organic acid and biomass material. **1% and *5%-levels of significant difference; ns, not significant at 5% level. Bars with different lowercase letters indicate a significant difference (p < 0.05) within one development stage. The same as **Figures 4** **–** **7**.

Some differences were noted in the response values of soil pH, electrical conductivity, and soluble salt content to the application of exogenous organic acids and biomass materials in different periods (**Figure 3**; **Supplementary Table S1**). Organic acids significantly affected soil electrical conductivity and soluble salt content at the seedling, elongation, and heading stages (*p* < 0.01), and biomass materials significantly affected soil electrical conductivity and soluble salt content at the three periods and soil pH at the elongation stage (*p* < 0.01). In addition, the interaction effect between organic acids and biomass materials highly significantly influenced soil electrical conductivity and soluble salt content in all periods (*p* ≤ 0.01) but not soil pH (*p* > 0.05). Soil pH in the CPN and CGC treatments was the lowest at the seedling stage and was not significantly different (*p* > 0.05) from that in the CCH, FPN, FGC, FCH, and CCM treatments but was significantly lower (*p* ≤ 0.05) than that in the remaining treatments. Soil electrical conductivity and soluble salt values were also significantly low (*p* < 0.05) in the CPN and CGC treatments. The soil pH in the CPN treatment was the lowest at the elongation stage and was not significantly different (*p* > 0.05) from that in the FPN, CCM, FCM, and FCH treatments, but it was significantly lower (*p* ≤ 0.05) than that in the remaining treatments. The soil electrical conductivity and soluble salt content in the CPN treatment were also significantly lower (*p* < 0.05) than those in the other treatments. Soil pH in the FPN, CPN, FCM, and CCM treatments was the lowest at the heading stage, and the difference between these treatments was not significant (*p* > 0.05). Soil electrical conductivity and soluble salt content in the FPN treatment were the lowest and were not significantly different from those in the FCM treatment (*p* > 0.05) but were significantly lower than those in the other treatments (*p* ≤ 0.05).

### Effects of different combinations of exogenous organic acids and biomass materials on soil nutrient content in tidal flats

**Figure 4:**
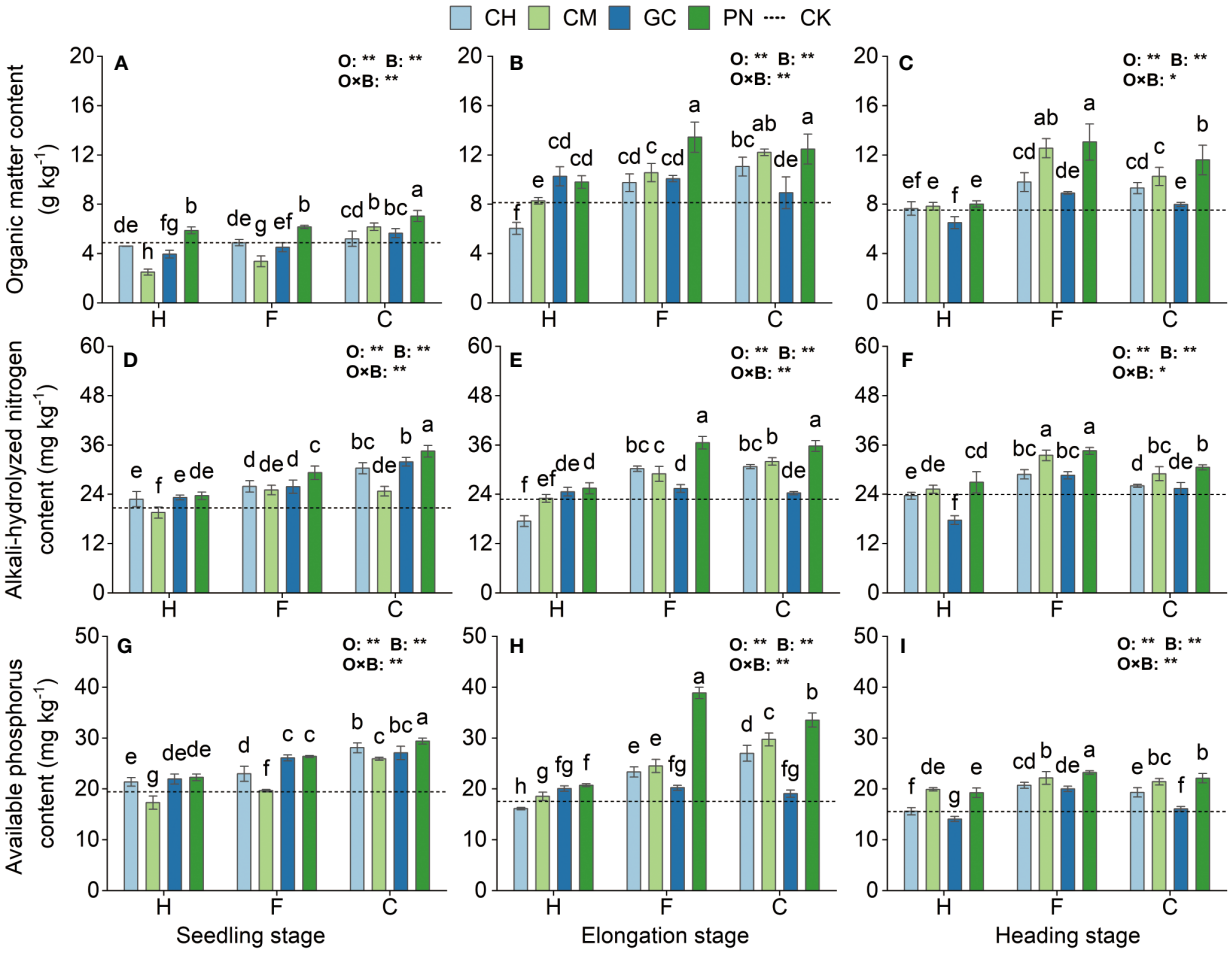
Effects of exogenous organic acids and substrates on the accumulation of organic matter, available phosphorus, and alkali-hydrolyzed nitrogen in mudflat soil at different periods. **(A, D, G)** Represent the seedling stage, **(B, E, H)** represent the elongation stage, and **(C, F, I)** indicate as the heading stage. **1% and *5%-levels of significant difference; ns, not significant at 5% level. Bars with different lowercase letters indicate a significant difference (p < 0.05) within one development stage.

Our results show highly significant differences (*p* < 0.01) in the response values of soil organic matter, available phosphorus, and alkali-hydrolyzed nitrogen content to the application of organic acids and biomass materials in the mudflat soils from the seedling stage to the heading stage (**Figure 4**; **Supplementary Table S1**). The interaction effects of organic acids and biomass materials reached a highly significant level (*p* < 0.01) for all indicators except soil organic matter and alkali-hydrolyzed nitrogen values at the heading stage, which were only significantly affected (*p* < 0.05). At the seedling stage, the soil organic matter, available phosphorus, and alkali-hydrolyzed nitrogen contents of the CPN treatment were significantly higher (*p* < 0.05) than those of the other treatments and increased by 44.17%, 66.73%, and 51.24%, respectively, compared with those of CK. Soil organic matter and alkali-hydrolyzed nitrogen contents in the FPN and CPN treatments were the highest at the elongation stage, and all indicators were significantly higher than those in the other treatments (*p* ≤ 0.05), except for soil organic matter, which was not significantly different from that in the CCM treatment (*p* > 0.05). The soil organic matter in the FPN treatment was the highest at the heading stage and was not significantly different from those in the FCM treatment (*p* > 0.05), but it was significantly higher than those in the other treatments (*p* ≤ 0.05). Soil alkali-hydrolyzed nitrogen values were also significantly high (*p* < 0.05) in the FPN and FCM treatments. Soil available phosphorus in the FPN treatment was also significantly higher (*p* < 0.05) than those in the other treatments at the elongation stage and heading stage.

### Effects of different combinations of exogenous organic acids and biomass materials on the morphological indexes and biomass of sweet sorghum

**Figure 5:**
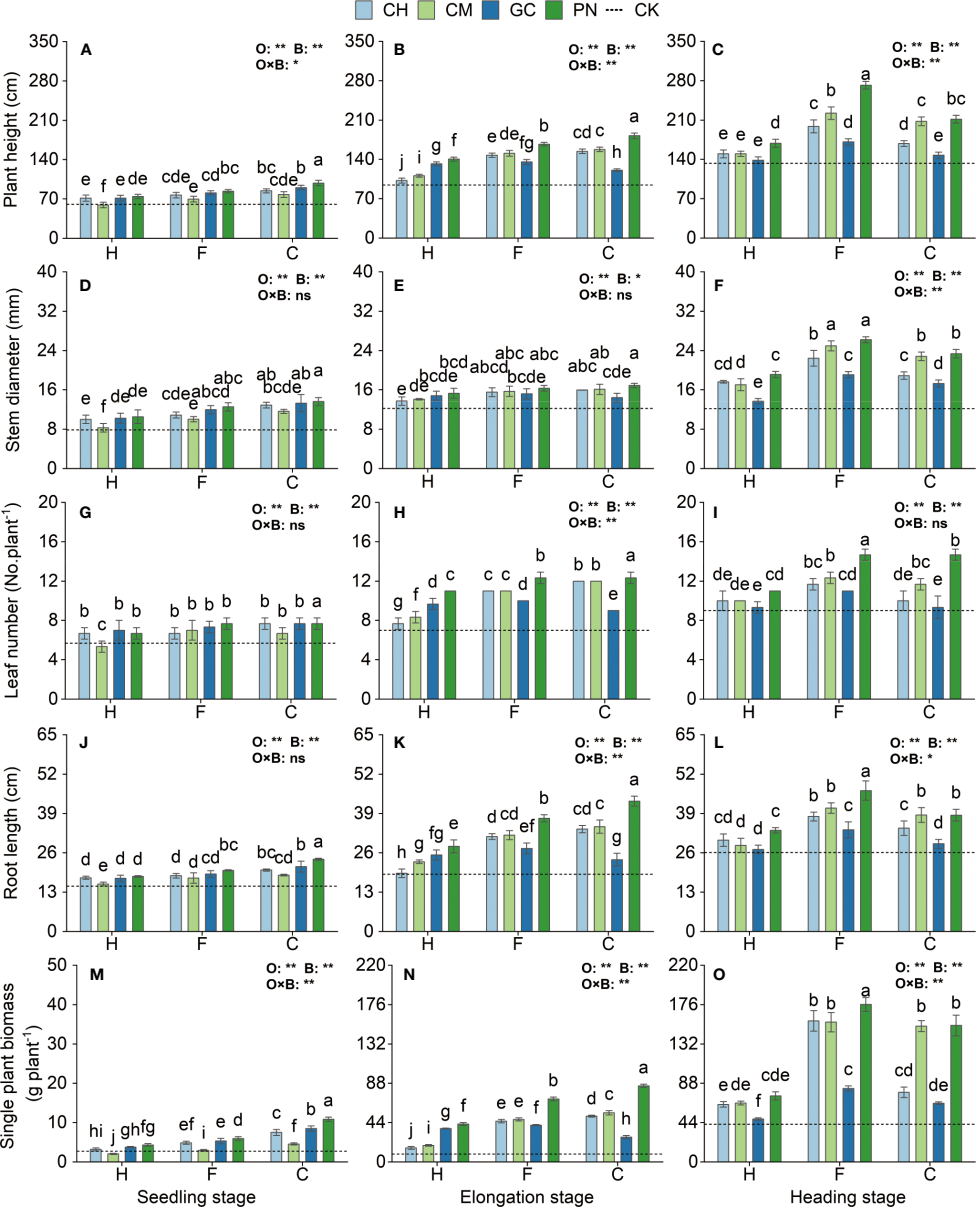
Effects of exogenous organic acids and biomass materials on the morphological indexes and biomass of sweet sorghum. **(A, D, G, J, M)** Represent the seedling stage, **(B, E, H, K, N)** represent the elongation stage, and **(C, F, I, L, O)** indicate as the heading stage. **1% and *5%-levels of significant difference; ns, not significant at 5% level. Bars with different lowercase letters indicate a significant difference (p < 0.05) within one development stage.

The morphological indicators and biomass of sweet sorghum differed in their responses to the application of exogenous organic acids and biomass materials at different growth stages (**Figure 5**; **Supplementary Table S2**). From the seedling to the heading stage, the plant height, stem diameter, leaf number, root length, and single plant biomass of sweet sorghum generally increased and showed highly significantly different changes (*p* < 0.01) in response to organic acids. Meanwhile, their responses to biomass materials also reached highly significant differences (*p* < 0.01), except for stem diameter, which only reached a significant difference level (*p* < 0.05) at the elongation stage. For the interaction effect between organic acids and biomass materials, a highly significant effect (*p* < 0.01) was observed on the plant biomass at the seedling stage, the plant height, leaf number, root length, and plant biomass at the elongation stage, and the plant height, stem diameter, and plant biomass at the heading stage; a significant effect (*p* ≤ 0.05) on the plant height at the seedling stage and the root length at the heading stage; and an insignificant effect (*p* > 0.05) on the other treatments. At the seedling and elongation stages, the plant height, stem diameter, leaf number, root length, and single plant biomass were higher in the CPN treatment than in the other treatments and increased from 62.71% to 93.08%, 38.41% to 73.63%, 58.73% to 85.72%, 59.35% to 209.11%, and 297.80% to 882.72%, respectively, compared with those in the CK. At the heading stage, the plant height, leaf number, root length, and plant biomass of the FPN treatment were significantly higher than those of the other treatments (*p* ≤ 0.05). The stem diameter of the FPN treatment also reached the highest but was not significantly different from that of the FCM treatment (*p* > 0.05). Each of the indexes increased by 104.23%, 318.56%, 63.00%, 78.16%, and 115.20% compared with those in the CK.

### Effects of different combinations of exogenous organic acids and biomass materials on the osmotic regulators, chlorophyll, and root activity of sweet sorghum

**Figure 6:**
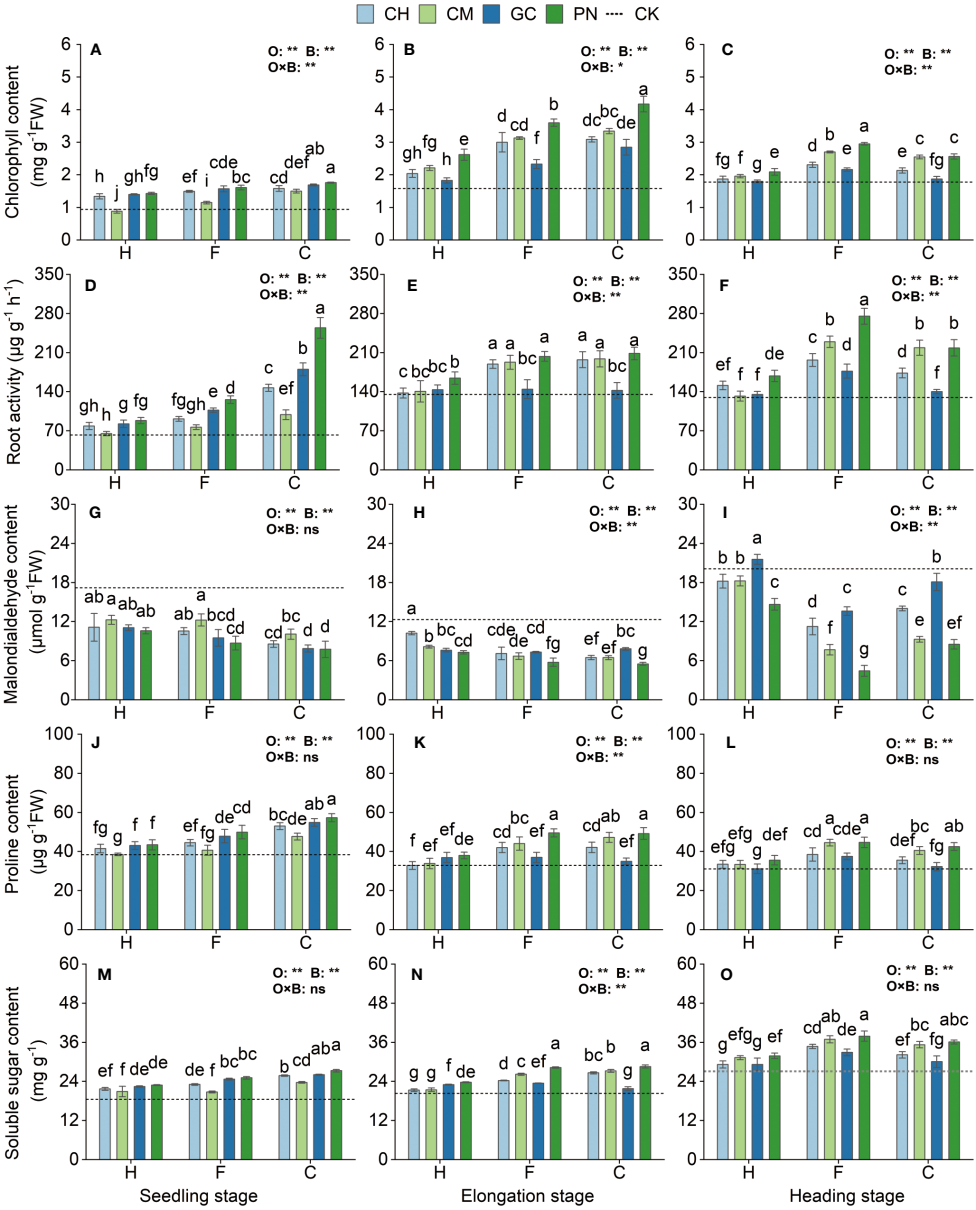
Effects of exogenous organic acids and biomass material on the osmotic regulation substances, chlorophyll, and root activity of sweet sorghum. **(A, D, G, J, M)** Represent the seedling stage, **(B, E, H, K, N)** represent the elongation stage, and **(C, F, I, L, O)** indicate as the heading stage. **1% and *5%-levels of significant difference; ns, not significant at 5% level. Bars with different lowercase letters indicate a significant difference (p < 0.05) within one development stage.

As shown in **Figure 6** and **Supplementary Table S3**, the response values of root activity, chlorophyll, malondialdehyde, proline, and soluble sugar of sweet sorghum to the application of exogenous organic acids and biomass materials reached highly significant levels (*p* < 0.01) from the seedling to the heading stage. The interaction effect of organic acids with biomass materials had a highly significant effect (*p* < 0.01) on root activity, and chlorophyll at the seedling stage, root activity, malondialdehyde, proline, and soluble sugar at the elongation stage, and root activity, chlorophyll, and malondialdehyde values at the heading stage; a significant effect (*p* ≤ 0.05) on chlorophyll at the elongation stage; and an insignificant effect (*p* > 0.05) on the other treatments. The CPN treatment had the highest chlorophyll, proline, and soluble sugar content at the seedling stage, followed by CGC; the difference between these two treatments was not significant (*p* > 0.05). Root activity in the CPN was the highest and was significantly higher than that in the other treatments (*p* < 0.05). Malondialdehyde content was significantly lower in the CPN, CGC, CCH, FPN, and FGC treatments than in the other treatments (*p* ≤ 0.05), but the differences were not significant (*p* > 0.05). Chlorophyll content was significantly higher (*p* ≤ 0.05) in the CPN treatment than in the other treatments at the elongation stage. Root activity in the CPN treatment was also the highest, but it was not significantly different (*p* > 0.05) from that in the FPN, CCM, FCM, CCH, and FCH treatments. Malondialdehyde content was significantly low (*p* ≤ 0.05) in the CPN and FPN treatments. Proline was the highest in the CPN treatment, but its values were not significantly different between FPN and CCM (*p* > 0.05) but were significantly different from those in the other treatments (*p* < 0.05). The soluble sugar values of the CPN and FPN treatments were significantly higher than those of the other treatments (*p* < 0.05). Chlorophyll and root activity were significantly higher (*p* < 0.05) and malondialdehyde content was significantly lower (*p* < 0.05) in the FPN treatment than in the other treatments at the heading stage. Proline and soluble sugar were the highest (*p* ≤ 0.05) in the FPN, FCM, and CPN treatments, but the differences among these three were not significant (*p* > 0.05).

### Effects of different combinations of exogenous organic acids and biomass materials on the antioxidant enzyme system of sweet sorghum

**Figure 7:**
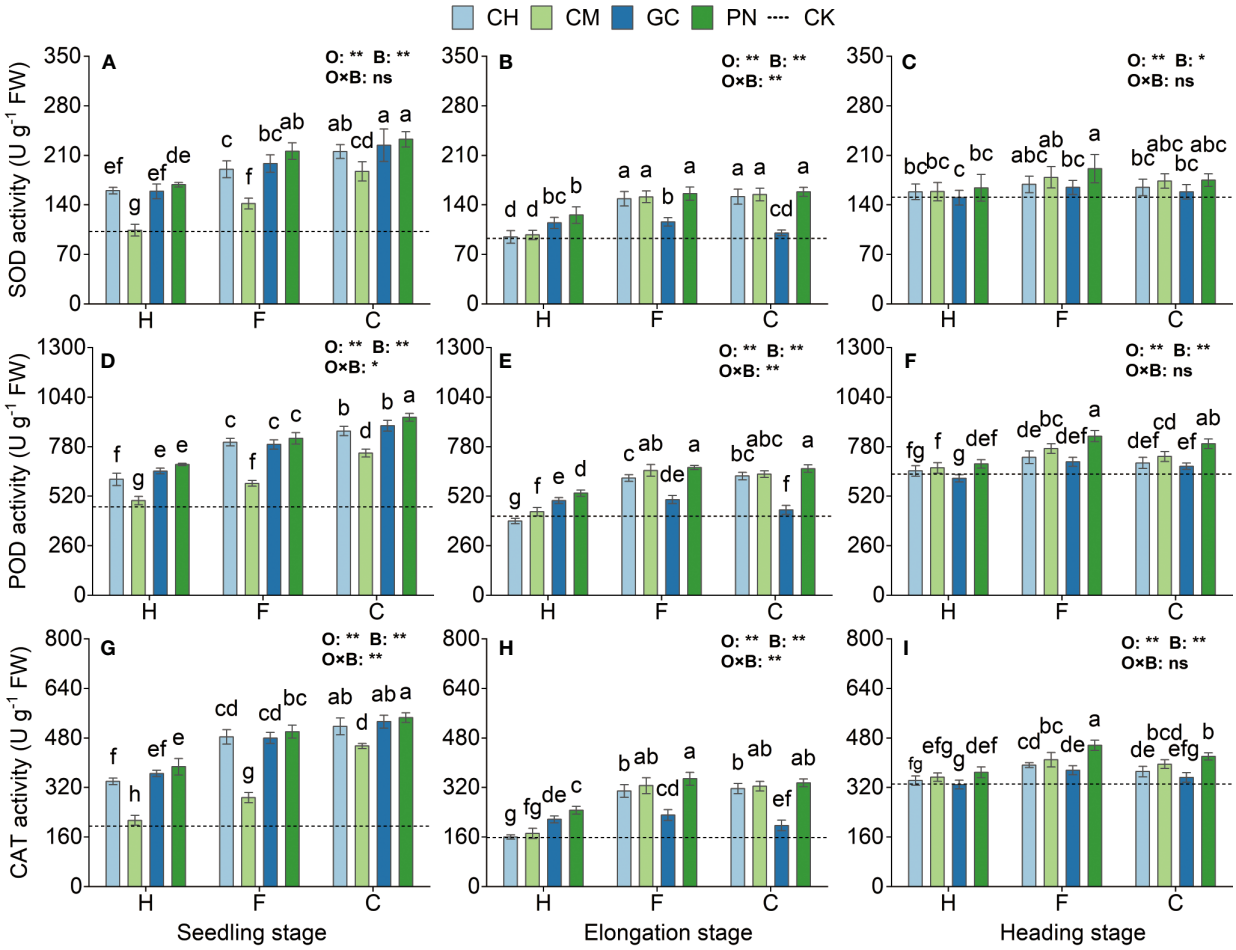
Effects of exogenous organic acids and biomass material on the antioxidant enzyme activities of sweet sorghum. **(A, D, G)** Represent the seedling stage, **(B, E, H)** represent the elongation stage, and **(C, F, I)** indicate as the heading stage. **1% and *5%-levels of significant difference; ns, not significant at 5% level. Bars with different lowercase letters indicate a significant difference (p < 0.05) within one development stage.

The SOD, POD, and CAT activities of sweet sorghum reached their highest levels at the seedling stage and decreased, followed by a small increase as the reproductive period progressed (**Figure 7**; **Supplementary Table S4**). Highly significant differences (*p* < 0.01) in SOD, POD, and CAT were observed in response to the organic acid treatment for sweet sorghum from the seedling to the heading stage. Except for SOD at the heading stage (*p* < 0.01), the response of other indexes to biomass materials was significantly different (*p* < 0.01). For the interaction between organic acids and biomass, extremely significant effects were observed on CAT at the seedling stage and on SOD, POD, and CAT at the elongation stage (*p* < 0.01); significant effects were also found on POD at the seedling stage (*p* < 0.05). SOD activity in the CPN and CGC treatments at the seedling stage was the highest; its value was not significantly different from that in the CCH and FPN treatments (*p* > 0.05) but was significantly higher than that in the other treatments (*p* ≤ 0.05). POD activity in the CPN treatment was significantly higher than that in the other treatments (*p* < 0.05). The CAT values in the CPN treatment were the highest; these values were not significantly different from those in the CGC and CCH (*p* > 0.05) but were significantly different from those in the other treatments (*p* ≤ 0.05). The SOD values of CPN, FPN, CCM, CCH, FCM, and FCH treatments were not significant differently from each other (*p* > 0.05) but were significantly higher (*p* ≤ 0.05) than those of the other treatments. For POD and CAT activities, both were the highest in the FPN, CPN, FCM, and CCM treatments at the elongation stage. The SOD activity of FPN, FCM, CPN, CCM, and FCH treatments at the heading stage was significantly higher than that of other treatments (*p* < 0.05). POD activity was the highest in the FPN treatment but was not significantly different from that in the CPN treatment (*p* > 0.05). CAT activity was significantly higher in the FPN treatment than in the other treatments during this period (*p* ≤ 0.05).

### Correlation analysis of soil improvement and sweet sorghum growth performance upon the application of combined exogenous organic acid and biomass

**Figure 8:**
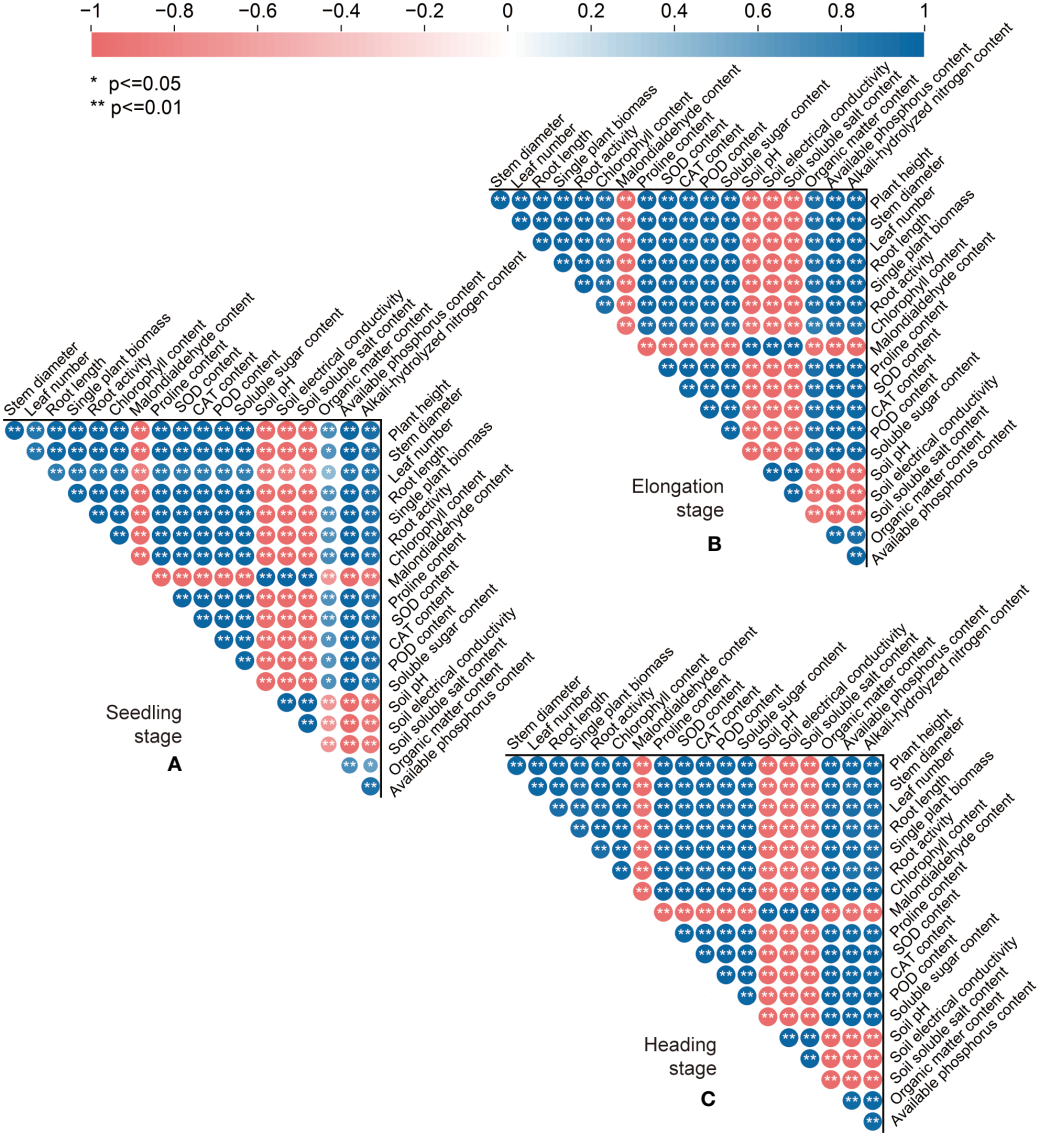
Spearman correlation analysis of the soil physical and chemical properties, physiological and biochemical characteristics, and growth indexes of sweet sorghum in different periods. **(A-C)** Represents the seedling stage, the elongation stage, and the heading stage, respectively. Blue indicates significant positive correlation, red indicates significant negative correlation, circle size indicates degree of correlation.

Spearman correlation analysis (**Figure 8**) showed that after the addition of exogenous organic acid and biomass materials, the biomass and morphological indexes (the plant height, stem diameter, leaf number, root length, and single plant biomass) of sweet sorghum in the three periods were significantly positively correlated with the physiological and biochemical indexes (the chlorophyll, proline, soluble sugar, SOD, CAT, and POD) of sweet sorghum (*p* ≤ 0.01), negatively correlated with the malondialdehyde content (*p* ≤ 0.01), positively correlated with soil nutrient indexes (the soil organic matter, available phosphorus, and alkali-hydrolyzed nitrogen) (*p* ≤ 0.05), and negatively correlated with soil saline–alkaline indexes (the soil pH, electrical conductivity, and soluble salt) (*p* ≤ 0.01). Meanwhile, the soil nutrient indexes were negatively correlated with soil salinity indexes (*p* ≤ 0.05), positively correlated with the physiological and biochemical indexes of sweet sorghum (*p* ≤ 0.05), and significantly negatively correlated with the malondialdehyde content (*p* ≤ 0.05).

## Discussion

### Processes and effects of treatment with exogenous organic acids and biomass materials on salinity regulation in coastal soils

The coastal mudflats in Jiangsu Province are affected by seawater groundwater and high temperatures in summer, and the phenomena of seasonal soil salting back and salt accumulation are serious. A large amount of sodium ions replace the calcium ions on the surface of the soil colloid, and the soil aggregate structure is destroyed, further aggravating the accumulation of salt in the soil. Organic acids are always undergoing a dynamic transformation of release, adsorption, and degradation in soils, but their sources are limited in alkaline soil environments, and their average levels have been low for a long time. The exogenous addition of organic acid-based biomass materials directly regulates soil acidity and alkalinity, promotes rapid soil desalination, and inhibits the upward transport of salts by forming a surface barrier (Mindari et al., 2018; Shan et al., 2022). For the three organic acids used in the current work, the soil treated with citric acid had lower soil pH, electrical conductivity, and soluble salt than those under other treatments at the seedling and elongation stages of sweet sorghum. This phenomenon probably occurred because citric acid is a ternary acid that can dissociate a large amount of H^+^ to neutralize basic ions and use anions to adsorb Na^+^ in the soil, thus reducing salinity (Santos et al., 2017). However, citric acid is highly soluble and melts, so maintaining its effect until the heading stage is difficult even with the stable solubility environment provided by the biomass material. Humic acid and fulvic acid are rich in active functional groups, such as carboxyl, hydroxyl, quinone, and amide groups, which can effectively increase the amount of cation exchange and regulate soil acid–base balance (Cui et al., 2022a; Vrantsi et al., 2021). However, fulvic acid has a relatively high active carbon group aliphatic chain structure, high saturation degree, stronger solubility, lower molecular weight, and stronger ion exchange force than humic acid, so its salt reduction ability is higher than that of humic acid (Guo et al., 2019; Sun et al., 2019). This study also found that among the four biomass materials, pine needles had stronger effects during different periods compared with cottonseed hull, grass charcoal, and cow manure. The possible reason is that the pine needle structure is rough and mostly consists of acicular thick leather, making the biomass less susceptible to rainfall leaching and soil biological interference. As a result, pine needles can effectively adsorb organic acids and have a slow effect on the soil. In addition, the weak acidity of pine needles can help reduce soil alkalinity, and the decomposition products can assist organic acids to exert chelation and eliminate soil salinity (Samec et al., 2021). Owing to its powdered structure and weak acidity, grass charcoal can adsorb organic acids during the seedling stage of sweet sorghum and rapidly infiltrate the active layer of the root system to chelate with soil saline components, effectively regulating the soil pore space through its own low bulk density to improve soil structure (Wang et al., 2020a; Yang et al., 2021b). However, the large specific surface area of grass charcoal leads to a reaction between a large number of organic acids immobilized by grass charcoal and the soil solution in the early stage, making it difficult to resist the upward movement and repeated accumulation of soil salt. Cow manure is rich in humus, which can improve the water leakage and fertilizer leakage characteristics of sandy soil. Its fibrous structure can cut the capillary continuity of the soil to reduce water and salt transport and assist the efficient water storage of the soil, effectively inhibiting the upward transport of soil salt under high-temperature conditions in the middle and late periods (El Sabagh et al., 2019). Cottonseed hull has a soft and flaky texture, a small bulk density, and high porosity, all of which are conducive to improving soil water and air conditions. However, its weak water retention hinders its effects when combined with organic acids (He et al., 2016).

### Regulatory effects of treatment with exogenous organic acids and biomass materials on the nutrient accumulation in coastal saline soils

Owing to their physicochemical and biological characteristics, saline–alkaline soils exhibit slow organic matter decomposition and low available nutrient stocks (Qadir and Schubert, 2002; Setia et al., 2013). As functional organic matter in the soil, high-molecular-weight humic acid and low-molecular-weight organic acids play an important role in soil morphology and fertility. Organic acids in soil are mainly derived from the decomposition of plant and animal residues, the secretion of plant roots, and the metabolism of microbes. Different types and concentrations of organic acids have varying effects on mineral dissolution and nutrient transformation in various kinds of soils. This study found that the exogenous application of citric acid, humic acid, and fulvic acid can increase soil organic matter, available phosphorus, and alkali-hydrolyzed nitrogen content. In particular, the effect of citric acid on sweet sorghum seedlings is stronger than that of fulvic acid. One reason is that the activation of difficultly soluble nutrients in the soil by organic acids relies on acid dissolution, anion complexing, and competitive adsorption; the acidity of organic acids and the number of dissociated anions determine their ability to activate nutrients (Li et al., 2014; Yang and Antonietti, 2020). Among the three organic acid treatments, citric acid has the strongest effect on phosphorus activation in alkaline soils, possibly because a large number of organic anions generated from citric acid dissociation dissolve the phosphorus that precipitated in the soil solution, aluminum, and iron; in addition, they compete for phosphorus adsorption sites in silicate minerals, effectively mobilizing the release of inorganic-bound nutrients (Wang, 2021; Wu et al., 2021b). Humic acid and fulvic acid are absorbent materials, and fulvic acid has more acidic active groups and a stronger adsorption effect on nitrogen and phosphorus than humic acid. Thus, fulvic acid effectively inhibits the transformation of activated nutrients into invalid states, continuously regulates the transformation of nutrient forms, and improves the long-term performance of nutrients, so its effect is highly significant in the middle and late stages. This finding is similar to the research results of Lv et al. (2022) and Yao et al. (2022b). The input of exogenous biomass directly increases the energy substrate of the beach soil, leading to a significant increase in the total amount of soil organic matter and nutrients. Holatko et al. (2022) and Sun et al. (2020) reported similar results. In the present study, pine needles significantly increased the contents of soil organic matter, alkali-hydrolyzed nitrogen, and available phosphorus over the whole growth period of sweet sorghum. The overall effect of cow manure was second only to that of pine needles, but the effect of cow manure at the seedling stage was not as prominent as that at the heading stage. A possible reason is that the weakly acidic pine needle material can provide a long-term suitable acid-base environment for soil nutrient activation. As leaf residues of plants, pine needles have more soluble nutrients than the other materials; their nitrogen and phosphorus elements exhibit a trend of first enrichment, then release, and gradually increasing, allowing them to permanently assist organic acid fertilization and directly increase soil nutrient content (Gao et al., 2019; Manral et al., 2020). The colloidal properties of cow manure can enhance the bonding of organic and inorganic complexes, promote the formation of soil aggregates and the accumulation of organic matter, and effectively enhance the availability and retention of nutrients (Chen et al., 2021a). However, its own nutrient content is relatively low, and the overall effect of combining the activation function of an organic acid with cow manure is slightly lower than that compared with pine needles. In addition, the improvement of soil nutrient content in the early stage is lower than that in the later stage. In summary, the physical and chemical properties such as surface area, pore size, and nutrient content of different biomass materials are important factors to consider in their application as excellent adsorption carriers of organic acids to enhance the improvement effect of organic acids on saline–alkaline soil (Hu et al., 2022; Islam et al., 2022).

### Mechanisms of the combination of exogenous organic acids and biomass materials to enhance salinity tolerance and biomass in sweet sorghum

During agricultural production, the high osmotic pressure in coastal saline soils caused by the high soluble salt content affects the kinetic and physiological–ecological processes of water and nutrient uptake by plant roots and decreases nutrient effectiveness in the soil, resulting in a long-term nutrient deficit in crops (Luo et al., 2017). Salinity stress and ion toxicity have negative effects on seed germination and root uptake, resulting in membrane lipid peroxidation, metabolic disorders, photosynthetic dysfunctions, and ultimately plant growth limitation and biomass decline (Tedeschi et al., 2011; Bhattarai et al., 2020). In this work, the addition of exogenous organic acids and biomass materials balanced the cellular osmotic potential by increasing the content of proline and soluble sugar content in plant leaves and the activity of some antioxidant enzymes used to scavenge reactive oxygen species. In addition, the accumulation of malondialdehyde was reduced to stabilize the plasma membrane, ensure the extension of the cell wall and normal cell growth, counteract the negative effects of salt ions on root activity and photosynthesis in aboveground parts, and promote biomass (Gulmezoglu and İzci, 2020; Yang et al., 2020). The added exogenous organic acids and biomass also reduced the uptake of salt ions by the root system by neutralizing and displacing saline components in the soil (Cui et al., 2022b) and stimulated the nutrient uptake and rapid growth of plants using the bioactive substances enriched in the material (Farid et al., 2017) and enhanced root vigor and aboveground photosynthesis (Chen et al., 2020; Zhang et al., 2020c), thus counteracting salinity stress by regulating root morphogenesis and cellular physiological processes (Olaetxea et al., 2016). The most significant effect of citric acid was found in sweet sorghum at the seedling and elongation stages, and the most significant effect of fulvic acid was found at the heading stage. This finding may be attributed to the different effects of exogenous organic acids on plant stems caused by their physicochemical properties (mainly acidity, interfacial activity, cation exchange capacity, complexation, adsorption, and dispersion) and type and amount of active functional groups (Shah et al., 2018). Citric acid has the highest number of carboxyl functional groups and is a low-molecular-weight organic acid that can be absorbed by plants. Its strong ligand affinity can effectively complex bond metal ions in soil or plants, quickly alleviate salt ion damage, and promote the growth of above-ground parts during the seedling stage (Tahjib-Ul-Arif et al., 2021). However, citric acid is easily leachable and has a short-duration fertilization effect, so it should be used in small amounts and at individual stages. By contrast, fulvic acid has the greatest variety of active functional groups. The number of its functional groups is higher than that of humic acid but less than that of citric acid. Fulvic acid has a strong buffering capacity, so its reaction time is slower than that of citric acid, but its effect is relatively long-lasting. It also promotes resistance enhancement and biomass accumulation in sweet sorghum (Braziene et al., 2021; Nargesi et al., 2022). When combined with the unique weak acidity and rough physical structure of pine needles, each of these two organic acids forms a salt barrier on the soil surface to reduce transpiration-induced salt transport to plant roots (Lu et al., 2012). The adsorbed organic acid functions for a long time under saline–alkaline stress by increasing plant root activity, enhancing the capacity of nutrient absorption and water retention, and reducing the osmotic stress and ion poisoning to ensure the stability of the plant photosynthetic system, maintain the physiological and biochemical reactions of sweet sorghum, and improve productivity.

### Effects of the combination of exogenous organic acids and biomass materials on the feedback system of “salt-tolerant forage grass—coastal saline soil”

The essence of the plant–soil feedback system is that plants and soil interact with each other through physical, chemical, and biological reaction mechanisms at different interfaces under different spatial and temporal patterns and scale levels, resulting in intricate positive and negative feedback effects and finally a coupled system with a bidirectional regulatory function formed by long-term evolution (Kandlikar et al., 2019). When plants are under stress conditions, signal substances secreted by roots act directly on soil or induce soil microorganisms to jointly regulate the soil physical structure and nutrient environment in the root zone, thus providing good water conditions and nutrient levels for aboveground parts (Vives-Peris et al., 2020; Chai and Schachtman, 2022). At the same time, the soil transmits information to plants when water and nutrients are at critical values, prompting plants to reduce their total nutrient uptake by changing their own traits or improving nutrient availability; this process generates a win–win effect through mutual feedback and regulation (Koster et al., 2019; Gong et al., 2020). Under saline–alkaline stress conditions, organic acids from different sources in the soil can be directly absorbed by roots and chelate salt ions in plants to convert them into binding states with relatively low activity, thus reducing the damage of salt ions to cells and strengthening the benign interaction between roots and soil (Jindo et al., 2020; Rakkammal et al., 2023). By complexing with the salts in the soil, organic acids reduce the direct contact and uptake of salts with plant roots, thus promoting root development, increasing the number of roots, and releasing a large amount of secretion to the soil in the root zone for positive feedback (Zanin et al., 2019). Among the three organic acids, citric acid had the strongest effect on soil through plants (Menezes-Blackburn et al., 2021). However, humic acid has more similarities in function and composition structure to the secreted substances from roots (Zhu et al., 2019), and their active components have similar effects on plant parts and rhizosphere soil. As plant growth regulators and soil conditioners, exogenous organic acids can directly stimulate or indirectly utilize the plant–soil feedback system to promote plant growth and physiological and biochemical reactions, regulate the soil environment, and enhance the strength of the interaction between salt-tolerant forage grass and coastal saline soil at the root surface and rhizosphere level. This treatment helps soil and plants resist salt invasion in the long term (Canellas et al., 2015). Biomass plays a role in the stable adsorption of exogenous organic acids into the soil and uses the unique water retention and nutrient metabolism characteristics given by their own physical and chemical structure; together with the function of organic acids, biomass also generates added value for the formation of the physical and chemical properties and microbial diversity of saline–alkaline soil (Abd El-Mageed et al., 2020; Abd El-Mageed et al., 2021). The effects of different organic acids and biomass materials on soil nutrient turnover and water–salt movement can lead to changes in the composition, structure, and function of soil microbial communities (Yao et al., 2021; Zhang et al., 2020a); such complex changes will be reflected back to plant productivity and the soil nutrient cycle through downward control (Lu et al., 2020; Tiwari et al., 2022). The process and role of soil microbial diversity in regulating the feedback of “salt-tolerant herbage—coastal saline–alkaline soil” will be further studied in the future.

## Conclusion

Exogenous organic acids and biomass materials and their interaction effects can affect the physicochemical properties of beach soils and the growth performance of sweet sorghum. Among the three organic acids used in this work, citric acid creates a beneficial soil environment for sweet sorghum under saline stress by stimulating its physiological activity and thus promoting its growth. Meanwhile, the slow-release nature of fulvic acid provides material for the long-term improvement of soil quality. Among the four biomass materials, pine needles can steadily maintain the effectiveness of the piggyback organic acids and continuously release organic acids and their own nutrients throughout all periods. Cow manure can effectively consolidate the effect of organic acid in the middle and late stages and assist in reducing salinity to promote crop growth; however, its ability to change soil is second only to a pine needle. In summary, citric acid–pine needle composite is suitable for harvesting short-term crops in coastal mudflats, and fulvic acid–pine needle or fulvic acid–cow manure composite can be used for long-term crops to improve the soil.

## Data availability statement

The raw data supporting the conclusions of this article will be made available by the authors, without undue reservation.

## Author contributions

ZS and RY conceived the ideas. RY and XLi collected the data. ZS and RY analyzed the data and led the writing. RY, YS, and XLo provided more arguments in the results and conclusion. ZS, YS, and LG proved the effectivity and rationality of the method proposed in this paper. All authors contributed critically to the ideas and drafts and gave final approval for publication.
